# Stress Hormone Dynamics Are Coupled to Brain Serotonin 4 Receptor Availability in Unmedicated Patients With Major Depressive Disorder: A NeuroPharm Study

**DOI:** 10.1093/ijnp/pyad041

**Published:** 2023-08-05

**Authors:** Gunild M Vulpius, Kristin Köhler-Forsberg, Brice Ozenne, Søren V Larsen, Arafat Nasser, Claus Svarer, Nic Gillings, Sune H Keller, Martin B Jørgensen, Gitte M Knudsen, Vibe G Frokjaer

**Affiliations:** Neurobiology Research Unit, Copenhagen University Hospital Rigshospitalet, Denmark; Psychiatric Center Copenhagen, Denmark; Neurobiology Research Unit, Copenhagen University Hospital Rigshospitalet, Denmark; Psychiatric Center Copenhagen, Denmark; Department of Clinical Medicine, University of Copenhagen, Denmark; Neurobiology Research Unit, Copenhagen University Hospital Rigshospitalet, Denmark; Department of Public Health, Section of Biostatistics, University of Copenhagen, Denmark; Neurobiology Research Unit, Copenhagen University Hospital Rigshospitalet, Denmark; Department of Clinical Medicine, University of Copenhagen, Denmark; Neurobiology Research Unit, Copenhagen University Hospital Rigshospitalet, Denmark; Neurobiology Research Unit, Copenhagen University Hospital Rigshospitalet, Denmark; Department of Clinical Physiology and Nuclear Medicine, Copenhagen University Hospital Rigshospitalet, Denmark; Department of Clinical Physiology and Nuclear Medicine, Copenhagen University Hospital Rigshospitalet, Denmark; Neurobiology Research Unit, Copenhagen University Hospital Rigshospitalet, Denmark; Psychiatric Center Copenhagen, Denmark; Department of Clinical Medicine, University of Copenhagen, Denmark; Neurobiology Research Unit, Copenhagen University Hospital Rigshospitalet, Denmark; Department of Clinical Medicine, University of Copenhagen, Denmark; Neurobiology Research Unit, Copenhagen University Hospital Rigshospitalet, Denmark; Psychiatric Center Copenhagen, Denmark; Department of Clinical Medicine, University of Copenhagen, Denmark

**Keywords:** Major depressive disorder, cortisol awakening response, SSRI, stress hormone dynamics, serotonin 4 receptor

## Abstract

**Background:**

A prominent finding in major depressive disorder (MDD) is distorted stress hormone dynamics, which is regulated by serotonergic brain signaling. An interesting feature of the cerebral serotonin system is the serotonin 4 receptor (5-HT_4_R), which is lower in depressed relative to healthy individuals and also has been highlighted as a promising novel antidepressant target. Here, we test the novel hypothesis that brain 5-HT_4_R availability in untreated patients with MDD is correlated with cortisol dynamics, indexed by the cortisol awakening response (CAR). Further, we evaluate if CAR changes with antidepressant treatment, including a selective serotonin reuptake inhibitor, and if pretreatment CAR can predict treatment outcome.

**Methods:**

Sixty-six patients (76% women) with a moderate to severe depressive episode underwent positron emission tomography imaging with [^11^C]SB207145 for quantification of brain 5-HT_4_R binding using BP_ND_ as outcome. Serial home sampling of saliva in the first hour from awakening was performed to assess CAR before and after 8 weeks of antidepressant treatment. Treatment outcome was measured by change in Hamilton Depression Rating Scale 6 items.

**Results:**

In the unmedicated depressed state, prefrontal and anterior cingulate cortices 5-HT_4_R binding was positively associated with CAR. CAR remained unaltered after 8 weeks of antidepressant treatment, and pretreatment CAR did not significantly predict treatment outcome.

**Conclusions:**

Our findings highlight a link between serotonergic disturbances in MDD and cortisol dynamics, which likely is involved in disease and treatment mechanisms. Further, our data support 5-HT_4_R agonism as a promising precision target in patients with MDD and disturbed stress hormone dynamics.

**Significance Statement**

A prominent finding in major depressive disorder (MDD) is distorted stress hormone dynamics, which is coupled to the cerebral serotonergic system. The serotonin 4 receptor is highly expressed in limbic brain structures and represents a promising novel antidepressant target. Establishing a link between stress hormone dynamics and the serotonin 4 receptor might define an important feature of disease and treatment mechanisms of MDD; however, it has not previously been investigated in patients with MDD. In this longitudinal study of patients with MDD, we show that serotonin 4 receptor availability is positively correlated with pretreatment cortisol awakening response. We believe our findings are potentially relevant for stratification and precision psychiatry care pathways of MDD by providing a plausible link between serotonergic disturbances and cortisol dynamics in MDD.

## Introduction

Major depressive disorder (MDD) is one of the most prevalent and debilitating psychiatric disorders, representing an enormous burden on patients, their relatives, and society. However, the pathophysiology of MDD is complex, multifactorial, and still poorly understood, and, accordingly, conventional antidepressant drugs targeting mainly the serotonin (5-HT) system largely vary in efficacy (Cipriani et al., 2018). Therefore, there is a need for relevant stratification of patients to facilitate targeted antidepressant prevention and treatment. A prominent feature in MDD that may inform such stratification is dysregulation of the hypothalamic–pituitary–adrenal (HPA) axis. Under normal circumstances, the HPA axis dynamically regulates cortisol output, which is critical for coping with both everyday life stress and more extreme stressors. It is cross-regulated with serotonergic brain function, with amygdala, hippocampus, and prefrontal cortex as important brain areas involved in this interaction (Bhagwagar et al., 2002; Lanfumey et al., 2008; Pruessner et al., 2010; Frokjaer et al., 2013). In MDD, dysregulation of the HPA axis, including cortisol dynamics, has repeatedly been found both in manifest depressive episodes and as a risk factor for developing depression (Bhagwagar et al., 2005; Stetler and Miller, 2005; Vreeburg et al., 2009; Adam et al., 2010; Booij et al., 2013; Stroud et al., 2019).

A useful biomarker for natural circadian cortisol dynamics is the cortisol awakening response (CAR), which refers to the transiently increased cortisol release and subsequent downregulation during the first hour after awakening (Pruessner et al., 1997). The CAR is readily accessible by home sampling of saliva, allowing for observation of cortisol dynamics in a natural setting without introducing stress. CAR has been proposed as a possible predicter of treatment outcome in MDD (Eikeseth et al., 2019; Refsgaard et al., 2022), and reestablishment of CAR is related to successful treatment with selective serotonin reuptake inhibitors (SSRIs) (Ruhé et al., 2015).

The postsynaptic serotonin 4 receptor (5-HT_4_R) can be imaged by positron emission tomography (PET) brain imaging in humans (Marner et al., 2009). Brain 5-HT_4_R density is particularly high in striatum and limbic regions, including hippocampus, and to a lesser extent in the cortex (Marner et al., 2010; Beliveau et al., 2017), which are brain regions thought to be both involved in MDD (Price and Drevets, 2010) and also in regulating the HPA axis (Pruessner et al., 2010). Moreover, we recently showed that untreated patients with MDD exhibit lower brain 5-HT_4_R binding compared with healthy controls (Köhler-Forsberg et al., 2023). It is also known that individuals at high familial risk for mood disorders have lower striatal 5-HT_4_R binding, suggesting it may be a trait factor in MDD (Madsen et al., 2015). Consistent with these studies, various evidence highlights 5-HT_4_R activation as a promising antidepressant target (for review, see Murphy et al., 2021). Importantly, preclinical studies have shown fast-acting antidepressant-like (Lucas et al., 2007) and anxiolytic-like (Mendez-David et al., 2014) effects of 5-HT_4_R agonism, and a recent study in healthy volunteers showed procognitive effects after a single dose of the 5-HT_4_R partial agonist prucalopride (Murphy et al., 2019). So far, no studies have investigated the antidepressant effects of 5-HT_4_R agonism or how it affects HPA axis regulation in patients with MDD, but recent evidence intriguingly shows that 5-HT_4_R agonism prophylactically decreases stress-induced depressive-like behavior in rodents (Chen et al., 2020). This suggests that compromised 5-HT_4_R signaling may be critically linked to maladaptation to stress, which would provide a plausible link between low 5-HT_4_R availability and depressed states (Köhler-Forsberg et al., 2023). We have previously demonstrated a negative association between CAR and 5-HT_4_R binding in neostriatum, prefrontal cortex, and anterior cingulate cortex in healthy volunteers (Jakobsen et al., 2016). However, we do not know if 5-HT_4_R binding is coupled to CAR in the depressed state or to what extent CAR changes with efficient pharmacological antidepressant treatment.

There were 3 aims of the present study. First, we investigated the relationship between brain 5-HT_4_R binding and CAR in a group of 66 untreated patients with moderate to severe depression (aim 1). We hypothesized that 5-HT_4_R binding in brain regions relevant for cortisol dynamics correlated with CAR. Also, we explored if CAR changed after 8 weeks of treatment with the SSRI escitalopram (aim 2), with the hypothesis that CAR increased as a sign of reestablished cortisol dynamics. Lastly, we investigated if pretreatment CAR predicted or was correlated with treatment outcome (aim 3).

## Materials and Methods

### Participants

We included data from the NeuroPharm 1 (NP1) study, which is a clinical, nonrandomized, naturalistic longitudinal study of predictors of antidepressant treatment outcome (Köhler-Forsberg et al., 2020). Upon inclusion, patients met the DSM-5 criteria for single or recurrent unipolar depression and had a total score >17 on Hamilton Depression Rating Scale 17 items (HAM-D_17_). Some highlighted exclusion criteria were (1) prior or present history of other Axis 1 psychiatric disorders, (2) severe somatic illness, and (3) use of pharmacological antidepressant treatment within 2 months before enrolment (Köhler-Forsberg et al., 2020). Baseline assessment included collection of cortisol data, HAM-D 6 items (HAM-D6) and HAM-D_17_ score, and PET and magnetic resonance (MR) brain imaging. Afterwards, patients started antidepressant treatment with the SSRI escitalopram in flexible doses from 5 to 20 mg, with the option of switching to duloxetine (serotonin-norepinephrine reuptake inhibitor [SNRI]) after 4 weeks, if needed. Patients were reassessed after 8 weeks to map changes in CAR and treatment outcome, defined as percent change in HAM-D_6_ total score from baseline to week 8 relative to baseline (Köhler-Forsberg et al., 2020).

Data were available from the Center for Integrated Molecular Brain Imaging database (Knudsen et al., 2016), containing data from 100 patients included in the NP1 cohort (Köhler-Forsberg et al., 2020). The 3 aims of the study were explored in 3 slightly different groups due to missing data. The group used for aim 1 consisted of 66 patients (68 patients had complete pretreatment CAR, of whom 2 had incomplete PET data). The group used for aim 2 consisted of 72 patients with complete CAR at baseline and/or week 8 (48 patients had complete CAR both at baseline and week 8, 20 had complete pretreatment CAR only, and 4 had complete CAR only at week 8). Of these 72 patients, 7 switched to duloxetine before week 8. The group used in aim 3 consisted of 66 patients (68 patients had complete pretreatment CAR, of which 2 dropped out before week 8). See supplementary Figure 1 in the supplementary Materials for additional details. All participants gave written informed consent. The study was registered at clinicaltrails.gov (NCT02869035) and was approved by the Committees of Health Research Ethics in the Capital Region of Denmark (protocol H-15017713), the Danish Data Protection Agency (04711/RH-2016-163), and Danish Medicines Agency (protocol NeuroPharm-NP1, EudraCT-number 2016-001626-34).

### The CAR

CAR was measured based on salivary home samples. Participant training, instructions, home-sampling procedures, storing, and cortisol analyses were carried out as previously described (Nasser et al., 2023). The home samples consisted of 5 serial measurements over the first hour from awakening (at 0, 15, 30, 45, and 60 minutes). CAR was computed as the area under the curve with respect to increase from baseline awakening cortisol (AUC_i_) (Pruessner et al., 2003). Salivary cortisol concentrations were determined by a chemiluminescence immunoassay method on the IDS-iSYS automatic analyzer (IDS PLC, Boldon, UK). The intra- and inter-assay variation of the method was <15%. At baseline, 3 participants had collected the cortisol samples during >70 minutes and 2 during <50 minutes from awakening. At week 8, two participants had collected the cortisol samples during >70 minutes. These samples were normalized by use of the following procedure:


$$\begin{array}{l} \frac{AUC_{i}}{x\;min}\cdot 60min \end{array}$$

where *x* is the reported time span of sample collection. Additionally, 1 participant had not indicated time of awakening at the baseline cortisol samples, so we set it to be 1 minute before the first cortisol sample.

### Brain imaging

PET and MR neuroimaging procedures are described in detail elsewhere (Hong et al., 2007; Köhler-Forsberg et al., 2020). Briefly, PET data were acquired by use of a high-resolution research tomography Siemens PET-scanner (CTI/Siemens, Knoxville, TN, USA) following an intravenous bolus of [^11^C]SB207145. All patients were scanned with a Siemens Prisma 3-Tesla scanner (Erlangen, Germany). T1-weighted MR images were aligned and co-registered to PET images. PET scans were motion corrected using the Air 5.2.5 method (Woods et al., 1992). Mean tissue time activity curves for grey matter volumes were extracted for kinetic modeling using the simplified reference tissue model with cerebellum (excluding vermis) as reference region (Marner et al., 2009). This yielded the nondisplaceable binding potential (BP_ND_) as the outcome measure for 5-HT_4_R binding. Pvelab software package was used for extraction of regions of interest (Köhler-Forsberg et al., 2020). The selected regions of interest were prefrontal cortex, anterior cingulate cortex, neostriatum, and hippocampus, as used in our previous work regarding 5-HT_4_R and CAR (Jakobsen et al., 2016), because these are known to be involved in regulation of the HPA axis or have high 5-HT_4_R density (Marner et al., 2010; Pruessner et al., 2010; Beliveau et al., 2017). In line with previous NP1 studies, we also assessed a “global” index of brain 5-HT_4_R BP_ND_ by use of a linear latent variable model (LVM) (Köhler-Forsberg et al., 2020).

### Statistics

#### Baseline CAR and 5-HT_ 4_R Binding in the Unmedicated Depressed State

In all aim 1 models, brain 5-HT_4_R BP_ND_ was log-transformed before modeling. The association between 5-HT_4_R BP_ND_ in the 4 brain areas and pretreatment CAR was evaluated using univariate multiple linear regression. We adjusted for age, sex, 5-HTTLPR genotype, severity of the depressive episode (measured by HAM-D_17_), and injected [^11^C]SB207145 mass per kg bodyweight. Age and sex were included because of their potential relation to 5-HT_4_R binding (Madsen et al., 2011a) and CAR (Stalder et al., 2016). 5-HTTLPR genotype was included because carriers of low-expressing genotypes (s-carriers) have been shown to have lower 5-HT_4_R binding (Fisher et al., 2012) and higher cortisol responses to stressors (Gotlib et al., 2008) compared with carriers of high-expressing genotypes (ll-carriers). Severity of the depressive episode was included because it may influence CAR (Pruessner et al., 2010; Refsgaard et al., 2022) and injected [^11^C]SB207145 mass per kilogram bodyweight as to account for potentially biasing 5-HT_4_R PET-measurements (Marner et al., 2010; Madsen et al., 2011b). Secondarily to the univariate analyses, we used an LVM to assess whether CAR was associated with a “global” index of brain 5-HT_4_R BP_ND_. The latent variable is a single “global” estimate of the shared correlation in 5-HT_4_R BP_ND_ in the selected brain areas (Köhler-Forsberg et al., 2020). The *lava* package in R was used to estimate the LVM (Holst and Budtz-Jørgensen, 2013).

Supplementary analyses were conducted to evaluate the robustness of the observed associations at baseline: (1) CAR was classified in a binary fashion (i.e., blunted vs nonblunted), and (2) women who used oral contraceptives (OC) were excluded to avoid potential confounding because recent evidence showed that healthy women who used OC exhibit 9%–12% lower 5-HT_4_R binding (Larsen et al., 2020) and blunted CARs (Høgsted et al., 2021) compared with those who do not use OC. We defined CAR categorically as blunted vs nonblunted, where the nonblunted response contained a positive AUC_i_ and a 50% minimum increase of AUC_i_ from baseline at awakening, calculated as:


$$\begin{array}{l} \frac{\left[cortisol\;peak\;\right]\left[cortisol\;baseline\right]}{\left[cortisol\;baseline\right]}*100\%. \end{array}$$

We first tested if sex interacts with CAR in a linear interaction model with 5-HT_4_R BP_ND_ as the dependent variable. Then, the association between 5-HT_4_R BP_ND_ and CAR was computed in a subgroup of individuals who did not use OC (i.e., men and women without any hormonal contraceptive use).

#### Longitudinal CAR and Treatment Outcome

For aim 2, the possible change in CAR across treatment was evaluated by use of a linear mixed model, using an unstructured covariance pattern for the residual variance. Using this model allowed to account for missing data in this group. For aim 3, we first tested if antidepressant treatment outcome, that is, change in HAM-D_6_ relative to baseline, was associated with pretreatment CAR in a multiple linear regression model adjusted for age and sex. Secondarily, we examined if pretreatment CAR could be used as a tool for predicting treatment outcome dichotomized as ≥50% or <50% change in HAM-D_6_ relative to baseline. By use of receiver operating characteristic (ROC) curves, we asked if a model including age, sex, and pretreatment CAR was a better predictor of dichotomized treatment outcome than a model with only age and sex. The ROC curve is interpretated by use of area under the curve (AUC), where an AUC of 0.5 indicates a predictive value of the model equal to chance and an AUC of 1.0 indicates a perfect predictive value of 100%. The 2 AUCs were then compared using an ROC test to assess whether their predictive values were significantly different.

For all analyses, 2-sided statistical tests were used, and *P < *.05 was considered statistically significant. Model assumptions were tested graphically by examination of the distribution of the residuals using qq-plots, and when there was deviation from normality, *P* values were computed using nonparametric bootstrap. Multiple comparisons were accounted for by use of max test adjustment (“single-step” option in R multcomp package) across all 4 tests in the univariate models (denoted *P*_*corrected*_). All statistical tests and graphical presentations were performed in R version 4.1.1 (R Core Team, 2022).

## Results

### Study Population

Demographic, clinical, PET, and cortisol measures are presented in Tables 1 and 2. Consistent with the prevalence of MDD, most patients were female (76% women, 50 out of 66). HAM-D_17_ median total score was 22 at baseline, corresponding to a moderate to severe depressive episode.

**Table 1.** Demographic, Clinical, PET and Cortisol Measures at Baseline

Parameter at baseline	Parameter value	n
Age (years), *mean ± SD (range)*	27 ± 7 (18–54)	66
Sex, *n (% of total group)*	Women: 50 (76%) Men: 16 (24%)	66
Hormonal contraceptive use^a^*n (% of total group)*	Users: 27 (41%) Non-users: 21 (32%) Missing (women): 2 (3%) Missing (men): 16 (24%)	66
BMI (kg/m^2^), *mean ± SD (range)*	25 ± 6 (17–45)	66
5-HTTLPR genotype, *n (% of total group)*	s-carriers: 43 (65%) ll-carriers: 23 (35%)	66
Type of depressive episode, *n (% of total group)*	Recurrent episode: 37 (56%) First episode: 29 (44%)	66
HAM-D_17_ total score, *median, IQR (range)*	22, 5 (18–31)	66
HAM-D_6_ total score, *median, IQR (range)*	12, 2 (7–15)	66
SB injected dose (MBq), *median, IQR (range)*	602, 37 (263–615)	66
SB injected mass per body weight, *(μg/kg)* *median, IQR (range)*	0.009, 0.007 (0.004–0.08)	66
Prefrontal cortex 5-HT_4_R BP_ND_, *mean ± SD (range)*	0.6 ± 0.1 (0.4–0.8)	66
Ant. cingulate cortex 5-HT_4_R BP_ND_, *mean ± SD (range)*	0.8 ± 0.1 (0.5–1)	66
Neostriatum 5-HT_4_R BP_ND_, *mean ± SD (range)*	3.5 ± 0.5 (2–5)	66
Hippocampus 5-HT_4_R BP_ND_, *mean ± SD (range)*	1 ± 0.2 (0.7–1.7)	66
Time between scan and CAR sample (days), *median, IQR (range)*	2, 2 (1–8)	66
CAR, (AUC_i_, nmol/L*min)^b^, *mean ± SD (range)*	208 ± 303 (−953 to 769)	66
Categorical CAR, *n (% of total group)*	Blunted: 19 (29%) Non-blunted: 47 (71%)	66
Time from wake-up to first cortisol sample (min)^c^ *median, IQR (range)*	1, 4 (0–5)	66
Time span of first 5 CAR parameters, (min)^b^ *median, IQR (range)*	60, 1 (57–66)	66

Abbreviations: 5-HTTLPR genotype, genotype of the serotonin transporter linked polymorphic region (s- or ll-carrier); AUC_i_, Area under the curve with respect to increase; BMI, body mass index; BP_ND,_ nondisplaceable binding potential; CAR, cortisol awakening response; HAM-D_6/17_: Hamilton depression rating scale 6/17 items; SB injected dose/mass per kilogram, injected dose/mass of [^11^C] labelled SB207145. ^a^Hormonal contraceptive users (women): 15 progesterone and estradiol pill, 4 progesterone only pill, 8 hormonal IUD; non-users: 20 without any contraceptive use, 1 copper-IUD; 2 missing.

^b^Including 5 normalized values (see Materials and Methods for further explanation).

^c^Including 1 estimated value (see Materials and Methods for further explanation).

**Table 2.** Demographic, Clinical, and Cortisol Measures at Week 8

Parameter at week 8	Parameter value	n
CAR (AUC_i_, nmol/L*min)^a^ mean ± SD (range)	179 ± 359 (−642 to 1287)	52^b^
Time from wake-up to first cortisol sample (min) median, IQR (range)	0, 3 (0–10)	52^b^
Time span of first 5 CAR parameters (min)^4^ median, IQR (range)	60, 0.5 (56–69)	52^b^
Treatment outcome (%) median, IQR (range)	60, 41 (−18 to 100)	66^c^
Categorical treatment outcome n (% of total group)	<50%: 16 (39%) ≥50%: 40 (61%)	66^c^

Abbreviations: AUC_i_, area under the curve with respect to increase; CAR, cortisol awakening response; Treatment outcome, % change in HAM-D_6_ at 8 weeks relative to baseline (a high percent change in HAM-D_6_ equals a better treatment outcome). ^a^Including 2 normalized values (see materials and methods for further explanation). ^b^Patient group with complete CAR at week 8. ^c^Patient group with complete CAR at baseline and treatment outcome measured at week 8.

### Baseline 5-HT_ 4_R Binding and CAR in the Unmedicated Depressed State

**Figure 1.**
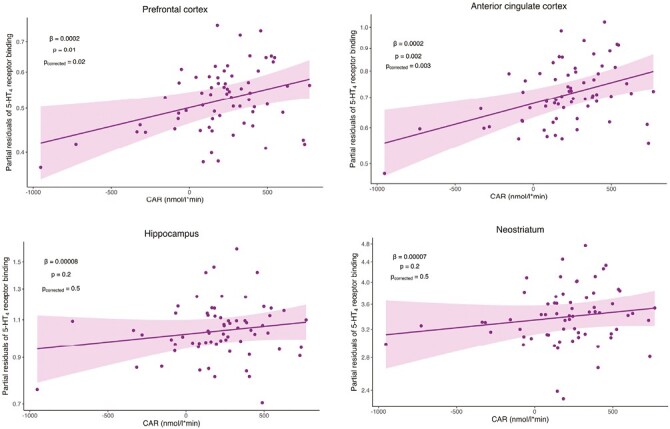
Scatter plot of the association at baseline between 5-HT_4_R binding (BP_ND_) and cortisol awakening response (CAR) adjusted for age, sex, 5-HTTLPR genotype, severity of depressive episode (HAM-D_17_ score), and [^11^C]SB207145 injected mass per kilogram. Shown in prefrontal cortex, anterior cingulate cortex, hippocampus, and neostriatum. 5-HT4R binding appears as it would if all participants were female s-carriers of the 5-HTTLPR gene with mean age, severity of depressive episode, and [^11^C]SB207145 injected mass per kilogram. Y scale is log transformed. Best fit line and 95% CI are indicated with line and shading.

5-HT_4_R BP_ND_ and CAR were significantly and positively associated in prefrontal cortex (*β *= .0002, 95% CI [0.00005; 0.0003], *P* = .01, *P*_*corrected* _= .02) and in anterior cingulate cortex (*β* = .0002, 95% CI [0.00008; 0.0003], *P* = .002, *P*_*corrected* _=_ _.003). The association was not significant in hippocampus (*β* = .00008, 95% CI [−0.00005; 0.0002], *P* = .2, *P*_*corrected* _ =_ _.5) or neostriatum (*β* = .00007, 95% CI [−0.00005; 0.0002], *P* = .2, *P*_*corrected*_ _ _=_ _ .5) (Figure 1). The LVM analysis did not show a significant correlation between global 5-HT_4_R BP_ND_ and CAR (γ = .00009, *P* = .1).

Supplementary analyses showed that the blunted group exhibited a significantly lower 5-HT_4_R BP_ND_ in prefrontal cortex (*β* = −.1, 95% CI [−0.2; −0.02], *P* = .02) and in anterior cingulate cortex (*β* = −.1, 95% CI [−0.2; −0.04], *P* = .006) (supplementary Figure 2). This is equivalent to 9.5% lower prefrontal and anterior cingulate cortices 5-HT_4_R BP_ND_ in the blunted compared with the nonblunted group. Further, we did not find an interaction between sex and CAR on 5-HT_4_R BP_ND_ in any of the brain regions (*P > *.5), and the positive association between 5-HT_4_R BP_ND_ and CAR remained significant in a subgroup consisting of 16 men and 21 women who did not use OC (prefrontal cortex: *β* = .0002, 95% CI [0.00006; 0.0004], *P* = .01) and anterior cingulate cortex: *β* = .0002, 95% CI [0.00006; 0.0004], *P* = .008).

### Longitudinal CAR and Treatment Outcome

**Figure 2.**
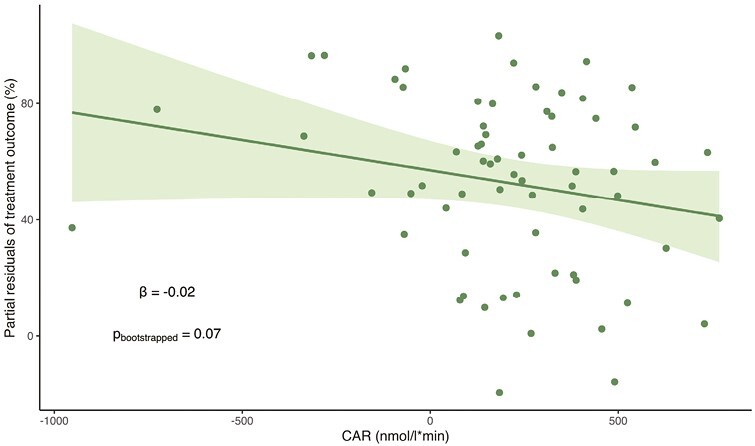
Scatter plot of the trend-level association between treatment outcome (percent change in HAM-D_6_ at week 8 relative to baseline) and pretreatment cortisol awakening response (CAR), adjusted for age and sex. Treatment outcome appears as it would if all participants were females with mean age. A high percent change in HAM-D_6_ equals a better treatment outcome. Best fit line and 95% CI are indicated with line and shading, and *P* value is derived using the percentile of the bootstrap distribution.

**Figure 3.**
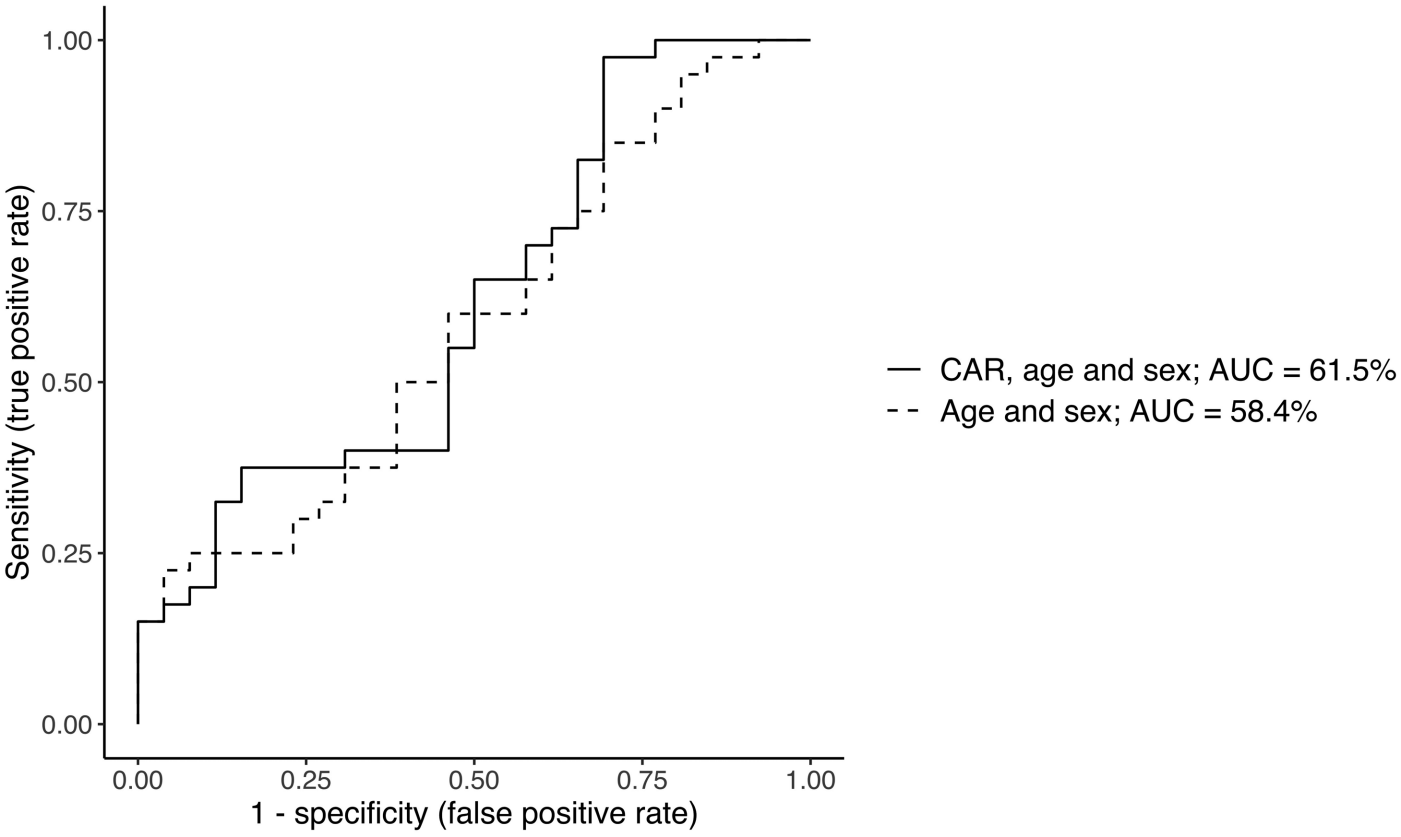
Receiver operating characteristic (ROC) curves illustrating pretreatment cortisol awakening response (CAR), age, and sex (solid line) and age and sex (dotted line) as possible predictors of antidepressant treatment outcome. Abbreviations: AUC = area under the curve. AUC = 0.5 indicates a predictive value of the model equal to chance and AUC = 1.0 a perfect predictive value of 100%.

The average pretreatment CAR did not significantly differ from the average CAR at week 8 (*β* = −28, 95% CI [−150; 94], *P* = .6). Treatment outcome was not significantly associated with pretreatment CAR; however, we observed a trend for a negative association (*β* = −.02, 95% CI [−0.05; 0.004], *P*_bootstrap_ = .07) (Figure 2). Pretreatment CAR did not have predictive power to distinguish a 50% or more reduction of depressive symptoms after 8 weeks: in the ROC curve including pretreatment CAR, age, and sex, the AUC was 61.5% (95% CI [47%; 76%]), which was not significantly better than the ROC curve with only sex and age as predictors of treatment outcome (ROC test: *z* = 0.8, 95% CI [−0.04; 0.1], *P* = .4) (Figure 3).

## Discussion

In this group of untreated patients with MDD, we demonstrate a positive association between prefrontal and anterior cingulate cortices 5-HT_4_R binding (BP_ND_) and cortisol dynamics in terms of CAR. The association is specific for those brain regions; it does not appear when assessing global brain 5-HT_4_R binding. We do not find that CAR changes after 8 weeks of antidepressant treatment, nor that pretreatment CAR can predict treatment outcome in terms of ≥50% or <50% improvement in symptom severity; however, it tends to be linked to the magnitude of change in depressive symptoms after 8 weeks of antidepressant treatment.

### Baseline 5-HT_ 4_R Binding and CAR in the Unmedicated Depressed State

The association between 5-HT_4_R binding (BP_ND_) and CAR demonstrated here supports a link between serotonergic brain function and cortisol dynamics in MDD. Interestingly, previous observations on CAR profiles in MDD are mixed, suggesting that both heightened and blunted CAR may be unfavorable in terms of risk for disease, treatment outcome, or relapse from remitted states. For example, heightened CAR is a prospective risk factor for the development of MDD (Adam et al., 2010), depressive symptomatology (Stroud et al., 2019), and risk of recurrence in patients with previous MDD (Hardeveld et al., 2014). At the same time, recent studies show that in patients with MDD, a blunted pretreatment CAR is associated with poor depressive symptomatology after 6 weeks and 6 months (Eikeseth et al., 2019) and after 2 years (Vreeburg et al., 2013). These mixed findings may, at least partly, be due to changes in CAR throughout the development and course of MDD, as demonstrated in previous studies showing heightened CAR in high-risk groups (Mannie et al., 2007; Vreeburg et al., 2010) and early stages of depressive episodes (Bhagwagar et al., 2005; Vreeburg et al., 2009), while blunted CAR has been found in later stages or chronic depression (Stetler and Miller, 2005; Booij et al., 2013). The loss of cortisol dynamics in severe or late stages of MDD could be explained by a loss of serotonergic control (Pruessner et al., 2010). This aligns with the notion that pharmacological serotonergic support may rescue depressed states partly via restoring cortisol dynamics and stress regulation capacities (Schüle, 2007; Ruhé et al., 2015). Intriguingly, our findings thus suggest that 5-HT_4_R agonism may also tap into such mechanisms. We know from observations in the same cohort that brain 5-HT_4_R binding is 7% lower in unmedicated patients with MDD than in controls (Köhler-Forsberg et al., 2023). Therefore, low prefrontal/anterior cingulate cortex 5-HT_4_R binding in the depressed state may be unfavorable in terms of compromised 5-HT4R signaling, which we speculate could lead to impaired control over cortisol dynamics. 5-HT_4_R agonism is a promising target in MDD, because rodent models suggest that direct stimulation of the 5-HT_4_R leads to rapid antidepressant effects (Lucas et al., 2007), appears to protect against stress (Chen et al., 2020), and is crucial for efficacy of SSRIs to rescue depressive- and anxiety-like behaviors (Mendez-David et al., 2014). Our data raise the question if 5-HT_4_R agonism supports cortisol dynamics in MDD and play a role in antidepressant treatment mechanisms.

Because the association between 5-HT_4_R binding and CAR demonstrated here is found only in prefrontal and anterior cingulate cortex, we speculate that prefrontal and anterior cingulate cortices 5-HT_4_R signaling comprise serotonergic top-down control over cortisol dynamics more than other brain areas. In support of prefrontal cortex involvement, prefrontal 5-HT_4_R stimulation upregulates the 5-HT firing rate in the dorsal raphe nucleus in mice (Lucas and Debonnel, 2002; Lucas et al., 2005; Faye et al., 2020), which can affect HPA axis dynamics via serotonergic brain signaling (Lanfumey et al., 2008). Serotonergic projections in hippocampus also regulate the HPA axis (Lanfumey et al., 2008; Pruessner et al., 2010), and recent evidence shows that hippocampal 5-HT_4_R signaling can modulate mood (Karayol et al., 2021). However, we do not see any association in hippocampus, possibly because of the confound of a reduced hippocampal volume in MDD (Price and Drevets, 2010).

Interestingly, 5-HT_4_R architecture appears to be different in the nondepressed population compared with patients with MDD; the present finding in MDD contrasts with our earlier observation in healthy volunteers, where we see a negative association between CAR and 5-HT_4_R binding (Jakobsen et al., 2016). This association in healthy individuals is evident in several brain areas supporting a more global effect. Interestingly, both human and rodent studies support that low 5-HT_4_R binding reflects higher synaptic serotonin concentration in the healthy state (Licht et al., 2009; Haahr et al., 2014). Thus, specifically in healthy individuals, low 5-HT_4_R binding may index higher serotonin tone, which is associated with healthy adaptations to stress and a robust CAR (Jakobsen et al., 2016). Meanwhile, low 5-HT_4_R availability may be disadvantageous in MDD. Further, the same opposite pattern of 5-HT_4_R associations with cognitive performance has been observed between MDD (Köhler-Forsberg et al., 2023) and healthy controls (Stenbæk et al., 2017). Taken together, these findings support the notion that 5-HT_4_R settings are altered in the depressed brain beyond what relates merely to adaptation to changes in synaptic serotonin.

### Longitudinal CAR and Treatment Outcome

Our longitudinal analysis shows that CAR is unaltered after 8 weeks of antidepressant treatment. This contrasts the only other study (to our knowledge) investigating changes in CAR before and after subchronic SSRI intake in patients with MDD, where an increase in CAR was coupled to remission (Ruhé et al., 2015). In this study, 2 salivary samples were used to calculate CAR, which mainly captures CAR peak timing rather than the whole trajectory of CAR (Stalder et al., 2016). We propose that this critical difference in CAR sampling may explain the discrepancy between the present finding and the former single study (Ruhé et al., 2015). In our study, 40 out of 66 patients achieved a ≥50% improvement in symptom severity 8 weeks from pretreatment. Thus, a lack of recovery cannot explain the absence of change in CAR, but without a placebo group, one cannot distinguish whether our finding is related to SSRI treatment or simply the natural course of symptomatology. In relation to this, 7 patients switched to duloxetine (SNRI) during the treatment period; however, our results remained unaltered in post hoc analyses excluding these 7 patients (supplementary Analysis 1). It may also be worth critically considering whether single-day measures of CAR at different time points are indeed a good measure of change in cortisol dynamics. Previous studies have shown substantial intra-individual variability in the CAR such that CAR on a single day to a larger extent is determined by situational factors rather than longer-term components. In future studies, this could possibly be accounted for by collecting cortisol samples at several days from every participant both before and after the intervention period (Hellhammer et al., 2007).

We do not find that pretreatment CAR is predictive of categorical treatment outcome in terms of ≥50% vs <50% improvement in symptom severity. We speculate that it may be too optimistic to expect a single measurement of CAR as a stand-alone clinically useful marker of treatment outcome, especially when considering the heterogenicity of patients with MDD and the multiplicity of factors leading to treatment response. This is also in line with previous findings suggesting that single predictors provide negligible improvements in predicting treatment outcomes (Beliveau et al., 2022; Dam et al., 2022; Fisher et al., 2022) while models combining a set of predictors may be more helpful (Zhou et al., 2022). However, pretreatment CAR tends to be linked to the magnitude of change in depressive symptoms such that lower pretreatment CAR is coupled to a better treatment outcome. On the same topic, 2 recent studies have shown significant correlations between CAR and treatment outcomes, albeit with opposing results. Importantly, none of these studies measure CAR in the unmedicated state; in both studies, a large proportion of patients already receive psychotropic medication at baseline [58% and 89%, respectively (Eikeseth et al., 2019; Refsgaard et al., 2022)]. Eikeseth et al. find that blunted CAR at baseline is linked to higher depressive severity after 6 weeks of psychotherapy, while Refsgaard et al. show that blunted CAR is linked to better treatment outcome after 9 weeks of SNRI treatment. To our knowledge, our study is the first to address longitudinal cortisol dynamics in patients who are unmedicated at baseline. The trend-wise association between pretreatment CAR and treatment outcome suggests that response to treatment including SSRI may be mediated, at least partly, through cortisol dynamics. This is in line with the hypothesis that optimizing cortisol dynamics in patients with MDD may be a tangible improvement in future stratification and precision psychiatry care pathways, which are greatly needed (Meehan et al., 2022).

### Methodological Considerations

Our study should be interpreted in the light of the following methodological considerations. First, inaccurate morning saliva sampling is suggested to be quite frequent and often associated with blunted CAR (Stalder et al., 2016). Because we did not objectively verify our CAR measurements, we cannot rule out inaccuracy of some CAR estimates. However, 29% of our patients display a blunted pretreatment CAR, which compares with what is generally expected from similar studies (Wüst et al., 2000). Second, the association between regional 5-HT_4_R binding and CAR was driven mostly by extreme observations, which indeed add to the power; however, if spurious, this could represent a false-positive finding. Notably, post hoc analyses without the 2 outliers showed similar effects but borderline significance in anterior cingulate and no significance in prefrontal cortex (supplementary Analysis 2). So far, no other dataset exists to cross-validate our findings, and replication in future studies is warranted.

Third, workday status is considered a possible confounder when assessing CAR (Stalder et al., 2016). We chose not to adjust for workday status, as we could not meaningfully discriminate between workday or day off in our patient group, where many patients were on sick leave or had altered work patterns. However, our results remain unaltered in post hoc analyses including workday status (supplementary Analysis 3). Fourth, in our analyses using PET outcomes, we included 2 participants with injected tracer mass per kg >0.064 μg/kg, which can potentially lead to mass dose effects and an underestimation of 5-HT_4_R binding (Madsen et al., 2011b). Notably, we corrected for injected mass, and our results remained unaltered when these patients were excluded from the analyses (supplementary Analysis 4).

## Conclusion

We speculate that brain 5-HT_4_R architecture is altered in MDD, such that low prefrontal/anterior cingulate 5-HT_4_R binding may be unfavorable in the depressed state because of reduced 5-HT_4_R stimulation and subsequently, a loss of control over cortisol dynamics. Here, we underline that targeting the 5-HT_4_R holds promise for optimizing clinical care and precision psychiatry for patients with depressive episodes, at least in patients with abnormal cortisol dynamics. Our longitudinal findings are less clear; pretreatment CAR does not predict treatment outcome; however, the 2 appear to be linked. Future studies should elucidate if 5-HT_4_R agonism may rescue disturbed cortisol dynamics in certain subgroups of patients with MDD.

## Supplementary Material

pyad041_suppl_Supplementary_Material

## Data Availability

The data underlying this article will be shared on reasonable request to the corresponding author.

## Interest Statement

Dr Frokjaer has served as consultant for Sage Therapeutics, H. Lundbeck and Janssen-Cilag. Prof. Knudsen is an expert advisor for Sanos, Onsero, PureTechHealth, Abbvie, and Gilgamesh and is a speaker for H. Lundbeck. She also has research contracts with Reunion and Delix Therapeutics. Prof. Jørgensen has given talks sponsored by Boehringer Ingelheim and H. Lundbeck. All other authors declare no conflict of interest.
