# Supplementary material for: Stress Hormone Dynamics Are Coupled to Brain Serotonin 4 Receptor Availability in Unmedicated Patients With Major Depressive Disorder: A NeuroPharm Study
Source: Int J Neuropsychopharmacol. 2023 Aug 5;26(9):639–48. doi: 10.1093/ijnp/pyad041 (PMC10519814; doi:10.1093/ijnp/pyad041)
Supplement: pyad041_suppl_Supplementary_Material [file pyad041_suppl_supplementary_material.docx]

**Supplementary Materials**

**
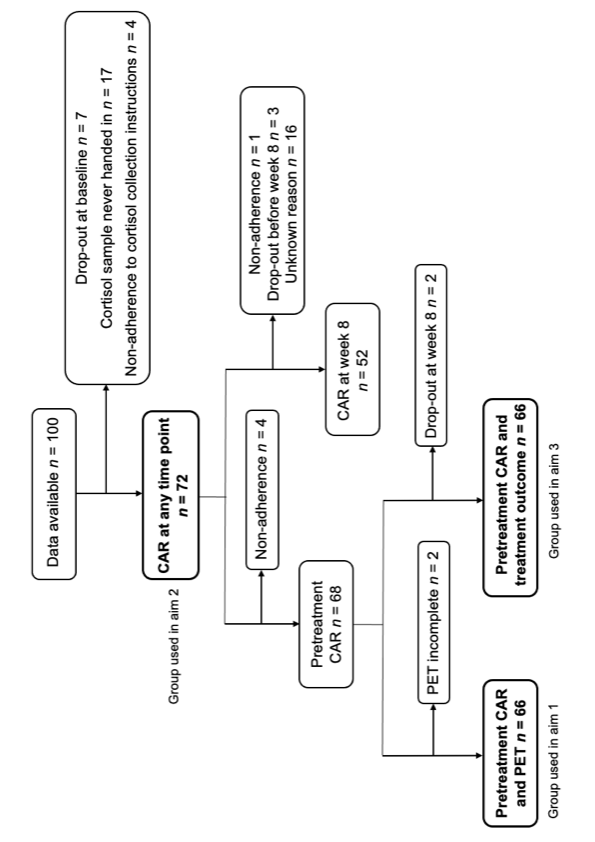
**

**Figure s1.** Flow chart of patient selection from the complete NP1 dataset.

**Figure s2.** Plot of 5-HT_4_R binding and dichotomized pretreatment cortisol awakening response (CAR) according to whether CAR is blunted or not. Adjusted for age, sex, 5HTTLPR genotype, severity of depressive episode (HAM-D_17_ score) and [^11^C]SB207145 injected mass pr kg. The association is shown in prefrontal cortex and anterior cingulate cortex. Y scale is log transformed. Mean 5-HT_4_R bindings are indicated by black squares.

**Analysis s1.** Linear mixed model investigating change in CAR from pretreatment to week 8, excluding 7 patients who switched to duloxetine before week 8 (*n* = 65):

*β* = -25, 95% CI [-155 ; 106], *p =* 0.7

**Analysis s2.** Multiple linear regression model investigating the association between 5-HT_4_R binding and pretreatment CAR without two outliers. Model adjusted for age, sex, 5-HTTLPR genotype, severity of depressive episode (HAM-D_17_ score) and [^11^C]SB207145 injected mass pr kg (*n* = 64):

*With CAR as a continuous variable:*

*Prefrontal cortex:* β = 0.0001, 95% CI [-0.00005 ; 0.0003], p = 0.16

*Anterior cingulate cortex:* β = 0.0002, 95% CI [-0.000004 ; 0.0003], p = 0.06

*With CAR as a categorical variable:*

*Prefrontal cortex:* β = -0.09, CI [-0.2 ; 0.01], p = 0.08

*Anterior cingulate cortex:* β = -0.1, CI [-0.2 ; -0.01], p = 0.03

**Analysis s3.** Multiple linear regression model investigating the association between 5-HT_4_R binding and pretreatment CAR. Model adjusted for age, sex, 5HTTLPR genotype, severity of depressive episode (HAM-D_17_ score), [^11^C]SB207145 injected mass pr kg and work day status (*n* = 57, 37 with day off and 20 with work day):

*Prefrontal cortex: β* = 0.0002, 95% CI [0.00002 ; 0.0003], *p =* 0.02

*Anterior cingulate cortex: β* = 0.0002, 95% CI [0.00005 ; 0.0003], *p =* 0.01

**Analysis s4.** Multiple linear regression model investigating the association between 5-HT_4_R and pretreatment CAR excluding two participants exceeding injected tracer mass/kg > 0.064 (*n* = 64). Model adjusted for age, sex, 5HTTLPR genotype, severity of depressive episode (HAM-D_17_ score) and [^11^C]SB207145 injected mass pr kg:

*Prefrontal cortex: β* = 0.0001, 95% CI [0.00002 ; 0.0003], *p =* 0.02

*Anterior cingulate cortex: β* = 0.0002, 95% CI [0.00008 ; 0.0003], *p =* 0.002
